# Accuracy of panoramic radiography, periapical radiography, and CBCT in diagnosing apical root resorption

**DOI:** 10.1590/1807-3107bor-2026.vol40.069

**Published:** 2026-08-24

**Authors:** Francieli Regina Bortoli, Filipe Colombo Vitali, Marcio Correa, Roberto Rocha, Fabrício Augusto Menegon, Leticia Fernanda Haas, Lucas da Fonseca Roberti Garcia, Cleonice da Silveira Teixeira

**Affiliations:** (a) Universidade Federal de Santa Catarina – UFSC, Department of Dentistry, Florianopolis, SC, Brazil.

**Keywords:** Cone-Beam Computed Tomography, Diagnostic Imaging, Radiography, Panoramical, Root Resorption

## Abstract

This study evaluated the accuracy of digital panoramic radiography, digital periapical radiography, and cone-beam computed tomography (CBCT) in diagnosing apical root resorption (ARR) using 85 extracted teeth from dry human skulls and mandibles. Four ARR levels were simulated: mild, moderate, severe, and extreme. CBCT scans, panoramic radiographs, and periapical radiographs were obtained before and after ARR simulation. Three examiners independently evaluated all images to determine ARR and, when present, to classify its severity level. Inter- and intra-observer agreement was calculated using Cohen's kappa, and sensitivity, specificity, and accuracy were determined for each method. Receiver operating characteristic (ROC) curve analysis was performed to obtain the area under the curve (AUC). AUCs were compared using the DeLong test. All imaging methods showed substantial inter- and intra-examiner agreement (κ > 0.60). CBCT demonstrated the highest sensitivity, specificity, and accuracy, followed by periapical and panoramic radiography. CBCT was significantly superior (p < 0.01) in detecting mild (AUC = 0.939) and moderate (AUC = 0.947) ARR compared with the other methods (AUC < 0.877). For severe ARR, CBCT (AUC = 0.953) and periapical radiography (AUC = 0.921) performed similarly (p > 0.05) and were superior (p=0.03) to panoramic radiography (AUC = 0.874). For extreme ARR, no significant difference was observed among the methods. In conclusion, CBCT showed high accuracy in detecting ARR, particularly at milder levels (< 2 mm). Periapical radiography was comparable to CBCT for severe resorption (one-third of the root), while all methods performed similarly for extreme resorption (half of the root). Thus, CBCT appears most suitable for early ARR detection, whereas periapical and panoramic radiography are adequate for diagnosing advanced stages.

## Introduction

Tooth root resorption is a complex biological process characterized by the irreversible loss of tooth structure, affecting the cementum, root dentin, or both layers.^1^ This condition can affect any region along the tooth root, with the apical region being the most susceptible.^2^ Primary etiological factors include pulp necrosis, trauma, and orthodontic movement.^2,3^ Among these, orthodontic treatment plays a significant role, as more than 80% of teeth under orthodontic forces exhibit apical resorption, and approximately one-third of patients experience a more severe loss exceeding 3 mm.^4-6^ Early diagnosis is challenging because the condition is typically asymptomatic, especially in teeth with vital pulp.^7^ Therefore, detection often relies primarily on radiographic examinations.^8,9^ Consequently, apical root resorption may progress unnoticed and be identified only once more advanced structural loss has occurred, complicating timely management.^8,9^

The selection of the most appropriate imaging modality for diagnosing apical root resorption remains inconsistent in the literature.^10^ Two-dimensional options, such as panoramic and periapical radiography, are widely available and remain the primary tools used in practice.^8,11^ However, these techniques have inherent limitations, including anatomical superimposition and the inability to visualize structural changes in three dimensions, which can hinder the detection of early resorptive changes.^8^ Conversely, cone-beam computed tomography (CBCT) provides three-dimensional imaging with greater anatomical detail, enabling the identification of apical changes that may not be visible on traditional radiographs.^9^ Although CBCT may offer diagnostic advantages in selected cases, these benefits must be weighed against higher radiation doses and increased costs.^10^ In addition, published studies present conflicting findings when comparing these modalities: some report clear benefits of CBCT for detecting early-stage resorption, whereas others demonstrate comparable performance between CBCT and periapical radiography, particularly when stricter diagnostic thresholds are applied.^10,12^

Given this variability in the literature, the diagnostic value of CBCT for apical root resorption remains uncertain. Reported sensitivity and specificity values range widely across studies, and methodological factors - such as differences in reference standards, image acquisition parameters, and the inherent difficulty of reproducing natural resorption patterns - further complicate direct comparisons. These inconsistencies highlight the importance of controlled experimental conditions to better delineate the diagnostic capabilities and limitations of each modality. Therefore, the present study aimed to evaluate the accuracy, sensitivity, and specificity of digital panoramic radiography, digital periapical radiography, and CBCT in diagnosing simulated apical root resorption. Furthermore, it aimed to evaluate the diagnostic performance of these imaging methods across varying levels of apical root resorption.

## Methods

### Sample selection

The present study was approved by the local Research Ethics Committee (protocol number 84.775.718). Five dentate, intact, dry, human skulls and mandibles were initially subjected to CBCT image acquisition to assess tooth eligibility. Mature permanent teeth presenting adequate alveolar bone support were selected. Exclusion criteria encompass teeth presenting incomplete root formation, root canal treatment, root caries or fracture, unusual root anatomy (e.g., apical bifurcation, severe dilaceration, or supernumerary root), periradicular lesions, evident external or internal root resorption, or vertical/horizontal alveolar bone loss.

The final sample comprised 85 human permanent teeth: 10 maxillary incisors, 10 maxillary canines, 10 maxillary premolars, 10 maxillary molars, 10 mandibular incisors, 10 mandibular canines, 10 mandibular premolars, and 15 mandibular molars. The number of teeth selected varied between skulls.

### Initial image acquisition (T1)

The dentate skulls and mandibles were subjected to the initial image acquisition (T1), which included panoramic, periapical, and CBCT scanning. The methods for obtaining the images are detailed below:

- *Panoramic radiography:* Images were obtained using the Cranex D X-ray machine (Soredex, Tuusula, Finland) operating at 57 kV and 10 mA (fixed), and a scan time of 11 seconds.^13^ During image acquisition, the skulls and mandibles were kept in position using adhesive tape to ensure stability. To ensure correct positioning within the focal trough, the skulls were aligned according to the manufacturer's panoramic positioning guidelines, using the unit's laser guides. The occlusal plane was positioned parallel to the floor, and the midsagittal plane was oriented perpendicular to the floor. Prior to each exposure, specimen alignment was visually verified to confirm proper positioning of the dental arches within the focal trough.
- *Periapical radiography:* Images were obtained using the parallel technique with Rinn XCP radiographic film positioners (Dentsply Rinn, Elgin, IL, USA). The positioners were stabilized by placing high-fusion Godiva impression compound (Lysanda, São Paulo, Brazil) between the teeth and the support, along with adhesive tape. Radiographic images were captured with the skulls and mandibles resting on a flat surface. A Spectro 70X Selectronic X-ray machine (Dabi Atlante, Ribeirão Preto, SP, Brazil) was used with a size 2 phosphor plates (Digora Optime, Soredex, Tuusula, Finland). The exposure parameters were 70 kV, 0.8 mA, and 0.25 seconds.^14,15^
- *CBCT scans:* CBCT scans were performed using an I-CAT scanner (Imaging Sciences International, Hatfield, USA) with an isotropic voxel size of 0.2 mm. The exposure parameters were 120 kV, 37.07 mA, and 26.9 seconds of scan time.^16-19^ The CBCT images were processed using Xoran software (version 3.1.62, Xoran Technologies, Ann Arbor, Michigan). The skulls and mandibles were stabilized during scanning using adhesive tape to maintain consistent positioning.

### Simulation of apical root resorption

A polyvinyl siloxane impression matrix was previously created to replicate the incisal edges of the anterior teeth and the occlusal surfaces of the posterior teeth. This matrix served as a guide to ensure the accurate repositioning of the extracted teeth into their original sockets after apical resorption simulation. Each experimental tooth was carefully extracted from its alveoli and examined using a magnifying glass (×4 magnification). Teeth were excluded if root cracks, fractures, resorptive processes, or excessive damage to the alveolar bone were identified.

The extracted teeth were randomly allocated into five groups, maintaining proportional representation within each tooth group, according to the simulated level of apical root resorption: level 0 (no resorption); level 1 (mild), irregular root contour; level 2 (moderate), resorption extending less than 2 mm from the apex; level 3 (severe), resorption involving approximately one-third of the root length – about 3 to 4 mm; and level 4 (extreme), resorption involving nearly half of the root length (> 5 mm).

**Figure 1:**
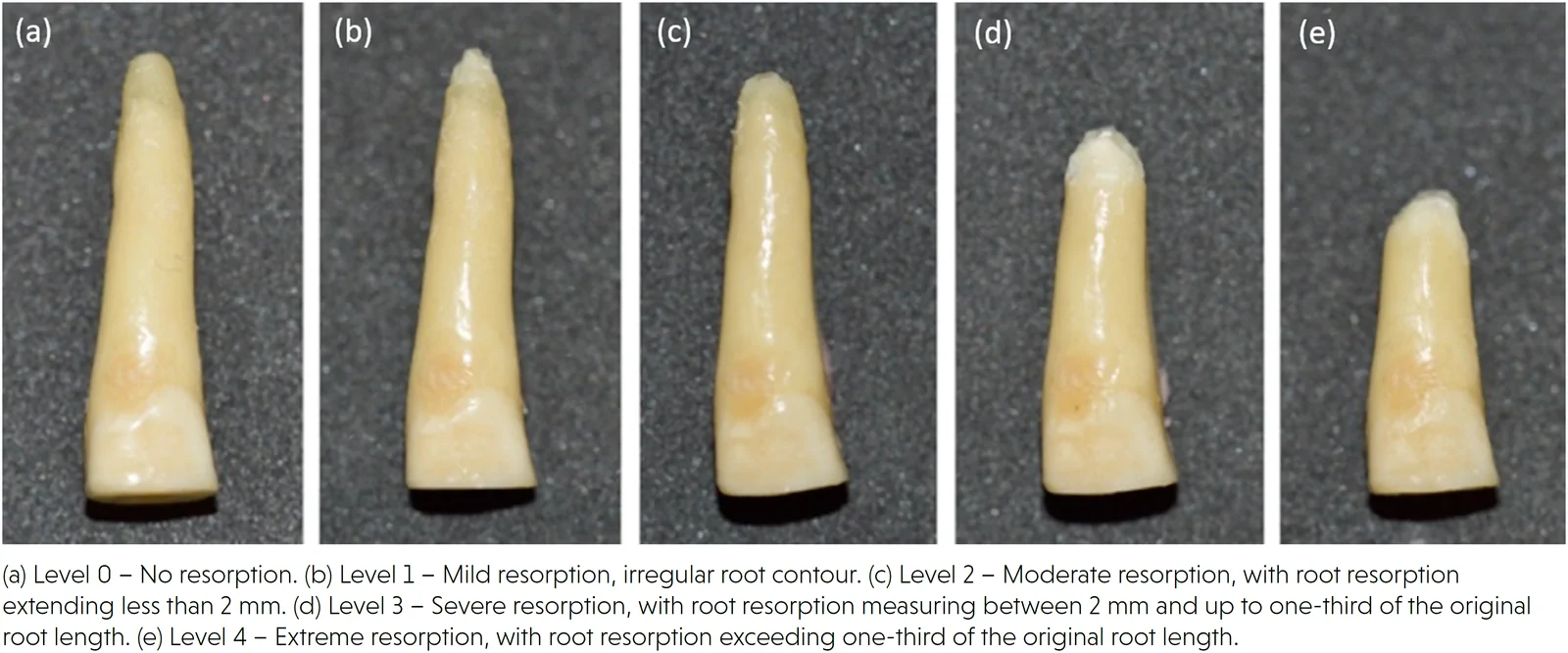
Illustrative images of apical root resorption levels following apical abrasion.

Figure 1 illustrates the levels of apical root resorption, as described by Levander and Malmgren.^20^ For the mild resorption simulation, apical abrasions were created using a #2200 diamond bur (KG Sorensen, São Paulo, SP, Brazil) to produce an irregular root contour. Moderate resorption was simulated by removing less than 2 mm from the apex using a conical stem diamond bur (#4138; KG Sorensen, São Paulo, Brazil). For severe and extreme levels, the root length was first measured using a dry-point compass and a millimeter ruler. Severe resorption was obtained by removing up to one-third of the root, and extreme resorption was simulated by removing up to half of the root length. All procedures were performed using diamond burs coupled to a high-speed handpiece, under copious air/water spray cooling, using an operating microscope (×8; DF Vasconcellos; Valença, Brazil).

Before tooth repositioning, the radicular portion of the teeth was uniformly coated with a 0.3-mm-thick layer of utility wax (Lysanda, São Paulo, Brazil) to achieve a radiographic appearance equivalent to the periodontal ligament space. In the skulls and mandibles, the sockets of teeth with simulated root resorption were filled with crushed bone, harvested from the skull using a bone crusher (Thimon, São Paulo, Brazil), to simulate bone tissue. Afterward, the teeth were repositioned into their sockets using the previously created matrix to ensure accurate alignment.

### Final image acquisition (T2)

**Figure 2:**
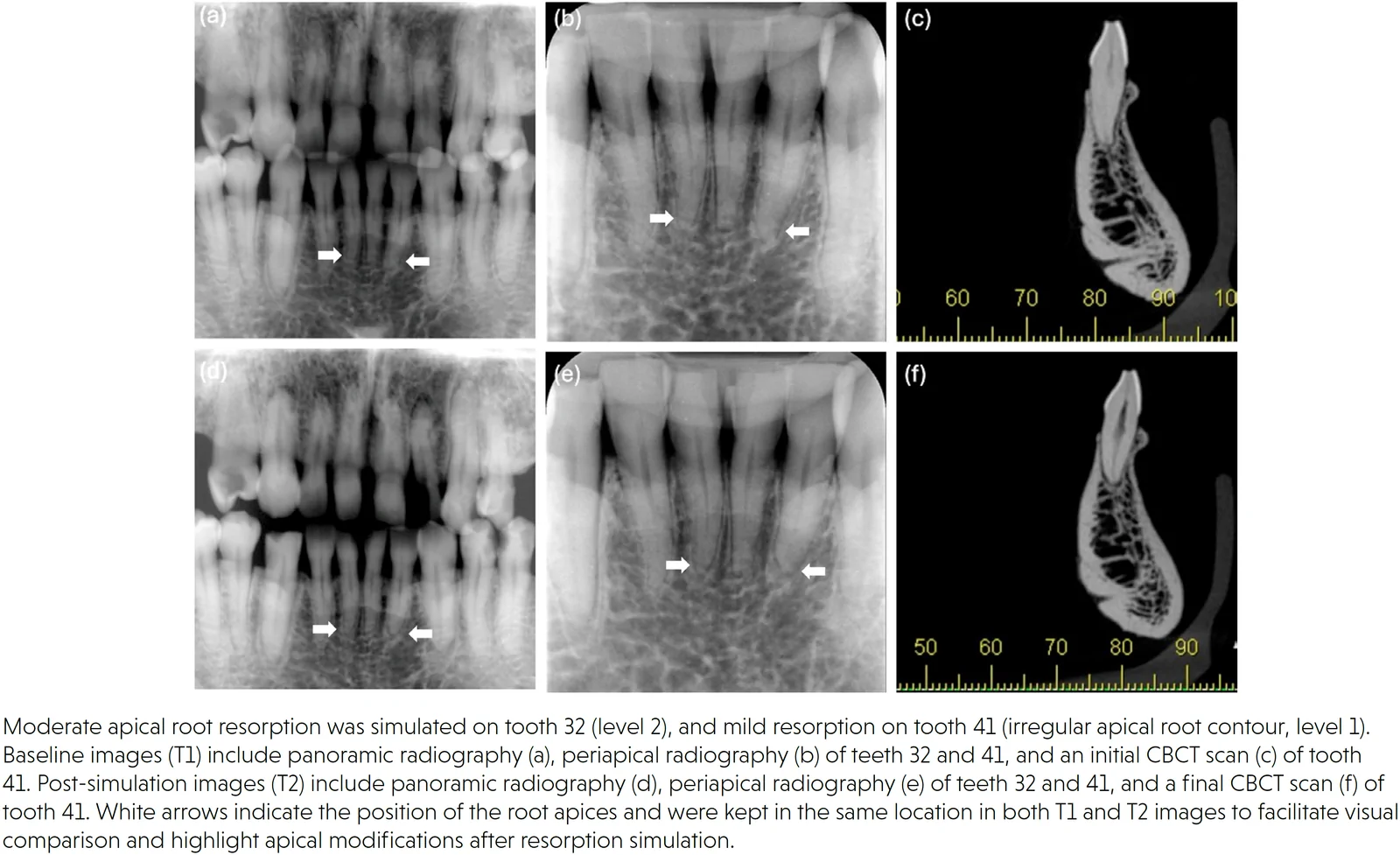
Representative images obtained before and after simulation of apical root resorption.

**Figure 3:**
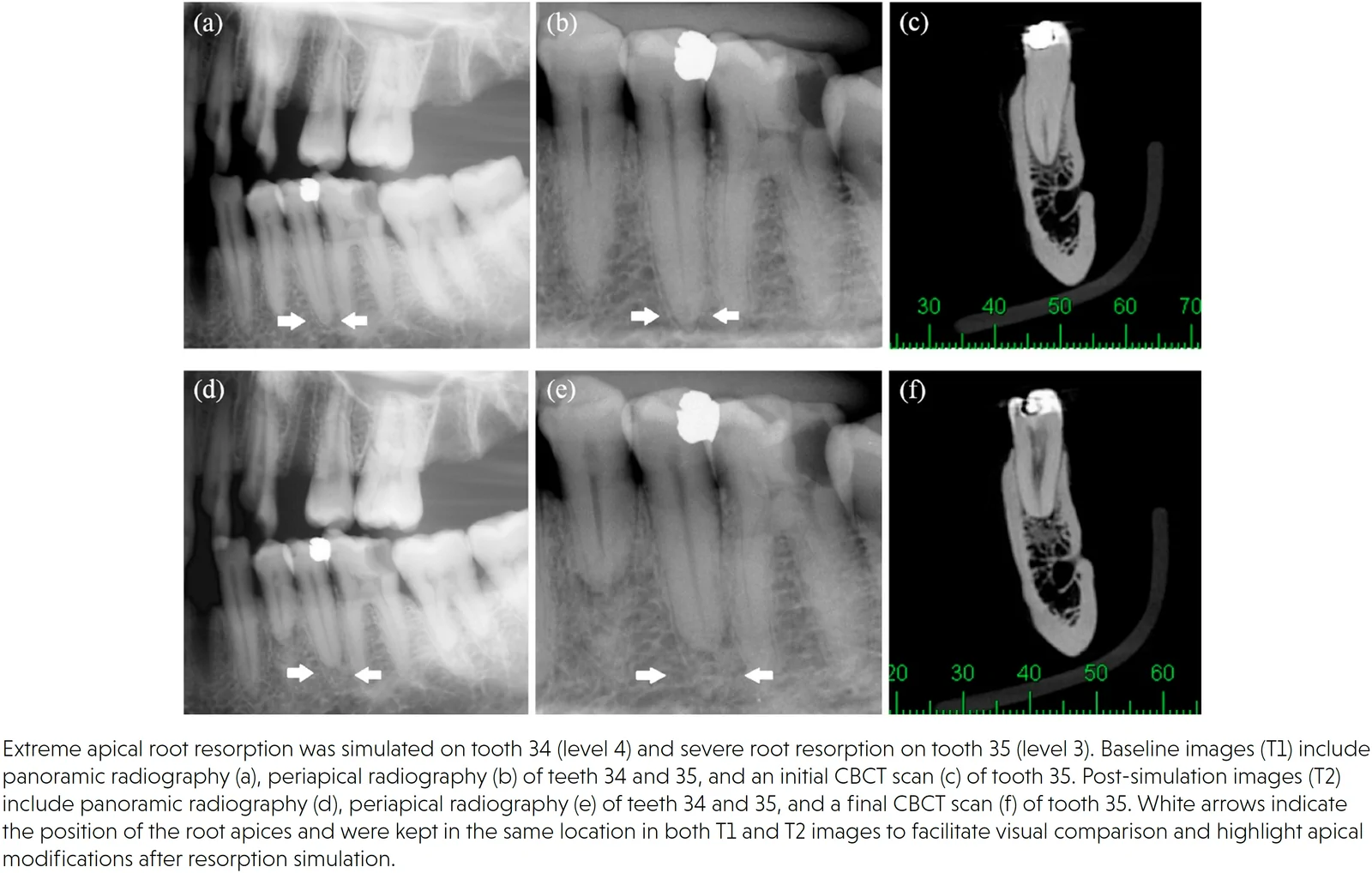
Representative images obtained before and after simulation of apical root resorption.

After repositioning the teeth in the skulls and mandibles, final image acquisition (T2) was performed using the same protocol used for the baseline images (T1). For CBCT evaluation, multiplanar reconstruction was performed using Xoran software (Xoran Technologies), and the synchronized views tool was used to match the same cross-sectional slice of each tooth between the initial and post-simulation scans. Anatomical landmarks guided slice selection to ensure consistent evaluation of the same root region, particularly the apical third. Due to inherent limitations in specimen repositioning and image reconstruction, exact slice matching was not always possible. Therefore, to standardize visualization of the resorptive areas, an experienced and blinded oral and maxillofacial radiologist performed minimal adjustments at T2, restricted to the same anatomical region of interest. These adjustments allowed consistent evaluation across all cases while preventing examiner-dependent variability. Figures 2 and 3 present images of the teeth before and after simulation of apical root resorption at different levels. Figure 2 presents mild and moderate resorption, whereas Figure 3 presents severe and extreme resorption, as visualized using the evaluated imaging methods.

### Image assessment

Three calibrated and blinded examiners (M.C., R.R., and C.S.T.) participated in image assessment. The examiners consisted of an oral and maxillofacial radiologist, an orthodontist, and an endodontist, each with over 20 years of experience in radiographic imaging analysis. For calibration, radiographs and CBCT scans of teeth exhibiting apical root resorption ranging from level 0 (no resorption) to level 4 (extreme root resorption) were presented to the evaluators, allowing them to discuss and align on the evaluation criteria.

The examiners individually assessed the images in six sessions, with a one-week interval between sessions. In each session, all 85 teeth were evaluated in T1 and T2 obtained images. Image assessment followed this sequence: sessions 1 and 4, panoramic radiography; sessions 2 and 5, periapical radiography; and sessions 3 and 6, CBCT scans. In each session, the level of apical root resorption was recorded in a spreadsheet using a five-point scale: (0) absent, (1) mild, (2) moderate, (3) severe, and (4) extreme. The digital images were evaluated on a 23-inch monitor with a 1920 x 1080-pixel resolution (Dell, Round Rock, USA), using imaging preview software (Xoran, version 3.1.62; Imaging Sciences International, Hatfield, USA) without a time limit for assessment. The digital tomography scans were evaluated in three reconstruction planes: axial, coronal, and sagittal. The size of the images was maintained at a 1:1 ratio; however, the examiners were allowed to enlarge the images by up to a maximum of 50%. For panoramic radiograph assessment, images with a 30% increase in brightness were also provided to improve visualization of the teeth in the anterior region. The images were arranged in a random and distinct sequence for each evaluation session. The number of the tooth to be examined was displayed to prevent any confusion.

### Statistical analysis

The data were analyzed using Stata software (version 17.0; Stata Corp., TX, USA) with a significance level set at 5%. Inter- and intra-observer agreements for each imaging modality were calculated using Cohen's Kappa test. The interpretation of the Kappa index followed the criteria established by Landis and Koch^21^. The mean and standard deviation of sensitivity (correct identification of apical root resorption), specificity (correct identification of the absence of resorption), accuracy (proportion of correct results), positive predictive value (PPV; probability of a true positive result), and negative predictive value (NPV; probability of a true negative result) were calculated for panoramic radiography, periapical radiography, and CBCT in detecting apical root resorption (absence versus presence). In addition, an ordinal receiver operating characteristic (ROC) curve analysis was performed to estimate the area under the ROC curve (AUC) for each imaging method, using direct visualization as the reference measure. This analysis was conducted at four distinct levels of resorption: level 1, level 2, level 3, and level 4. The AUC was calculated for each method to assess their accuracy in identifying resorption at these different levels. In each analysis, AUCs were compared using the DeLong test. If a significant difference was identified (*p* < 0.05), pairwise comparisons were conducted using the DeLong test to determine where significant differences occurred.

## Results

Table 1 presents the inter- and intra-examiner agreement in detecting apical root resorption using digital panoramic radiography, digital periapical radiography, and CBCT image. The Cohen's Kappa test showed substantial inter-examiner agreement (K > 0.60) across all imaging modalities. Intra-examiner reproducibility revealed Kappa scores greater than 0.60 for all evaluators with panoramic and periapical radiographs, indicating substantial agreement. For CBCT images, two evaluators achieved excellent agreement (K > 0.80), while one demonstrated good agreement (K = 0.69).

**Table 1:** Kappa test values (K) and corresponding 95% confidence intervals (CI) for inter- and intra-examiner agreement in detecting apical root resorption using digital panoramic radiography, digital periapical radiography, and CBCT image.

Variable		Panoramic radiography	Periapical radiography	CBCT image
Inter-examiner		0.62 (0.51–0.72)	0.71 (0.60–0.82)	0.79 (0.67–0.90)
Intra-examiner				
	Examiner 1	0.61 (0.48–0.70)	0.66 (0.55–0.76)	0.81 (0.69–0.91)
	Examiner 2	0.64 (0.53–0.75)	0.64 (0.52–0.67)	0.69 (0.57–0.79)
	Examiner 3	0.61 (0.50–0.72)	0.76 (0.65–0.87)	0.86 (0.76–0.97)

CBCT: cone beam computed tomography.

The sensitivity, specificity, accuracy, PPV, and NPV for digital panoramic radiography, digital periapical radiography, and CBCT image in detecting apical root resorption are summarized in Table 2. CBCT demonstrated high sensitivity in detecting apical root resorption, correctly identifying 94.4% of resorption cases. It also showed superior performance in identifying cases without resorption, with a specificity of 0.920. Consequently, CBCT exhibited an overall accuracy of 0.937, reflecting the high proportion of correct results. Digital periapical and panoramic radiography followed, with accuracy values of 0.898 and 0.847, respectively. When resorption was diagnosed, the probability of it being a true positive (PPV) was greater than 92% for all three imaging modalities. Conversely, if resorption was not detected, the probability of it being a true negative (NPV) was 87% for CBCT, 80.2% for digital periapical radiography, and 70% for digital panoramic radiography.

**Table 2:** Mean and standard deviation of sensitivity, specificity, accuracy, positive predictive value (PPV), and negative predictive value (NPV) for digital panoramic radiography, digital periapical radiography, and CBCT image for apical root resorption detection.

Variable	Sensitivity	Specificity	Accuracy	PPV	NPV
Panoramic radiography	0.850 (0.092)	0.840 (0.073)	0.847 (0.083)	0.927 (0.088)	0.700 (0.078)
Periapical radiography	0.911 (0.093)	0.867 (0.080)	0.898 (0.071)	0.942 (0.082)	0.802 (0.073)
CBCT image	0.944 (0.075)	0.920 (0.079)	0.937 (0.067)	0.967 (0.079)	0.873 (0.060)

CBCT: cone beam computed tomography.

**Figure 4:**
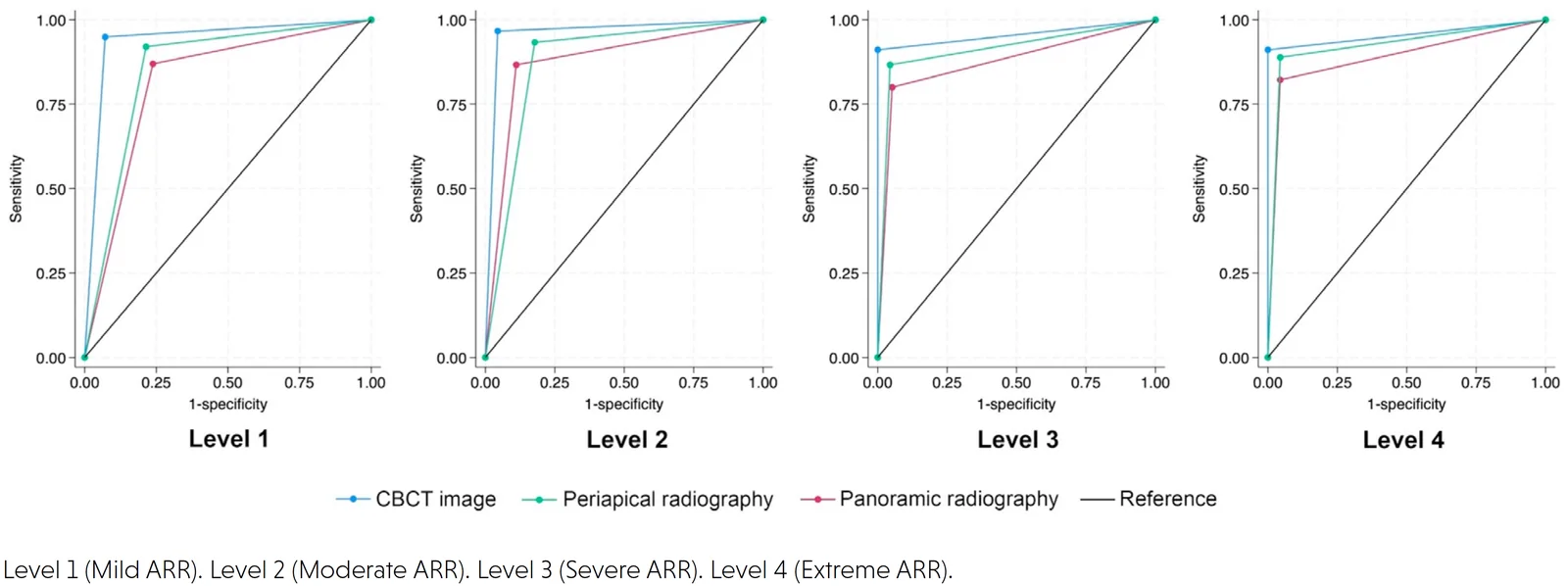
Receiver operating characteristic (ROC) curve for each imaging method in detecting four distinct levels of apical root resorption (ARR), using direct visualization as the reference.

Table 3 presents the AUCs for each imaging method, using direct visualization as the reference standard and considering an ordinal evaluation of the levels of apical root resorption. The results indicate that the CBCT image exhibited a significantly greater ability to diagnose mild (p < 0.01) and moderate (p < 0.01) of apical root resorption compared to digital periapical and panoramic radiography. For severe resorption, no significant difference (p > .05) was observed between CBCT and periapical radiography. However, both modalities demonstrated greater diagnostic capacity (p = .03) than panoramic radiography for severe resorption. For extreme resorption, no statistical difference was observed among the imaging methods (p = 0.07). Figure 4 shows the ordinal ROC curves considering each level of apical root resorptions evaluated.

**Table 3:** Area under the ROC curves and standard deviation for digital panoramic radiography, digital periapical radiography, and CBCT image in detecting different levels of apical root resorption using direct visualization as the reference standard.

Variable	Level 1	Level 2	Level 3	Level 4
	Mild	Moderate	Severe	Extreme
Panoramic radiography	0.815 (0.036)^A^	0.873 (0.025)^A^	0.874 (0.031)^A^	0.889 (0.030)^A^
Periapical radiography	0.853 (0.034)^A^	0.877 (0.024)^A^	0.921 (0.027)^B^	0.925 (0.025)^A^
CBCT image	0.939 (0.022)^B^	0.947 (0.009)^B^	0.953 (0.021)^B^	0.956 (0.021)^A^
p-value*	< 0.01	< 0.01	.03	0.07

* DeLong test.

CBCT: CBCT: cone beam computed tomography.

## Discussion

Apical root resorption resulting from orthodontic treatment is a relatively common occurrence.^3,5^ Since it is typically asymptomatic, diagnosis relies on radiographic examinations, with panoramic and periapical radiographs being the most frequently used methods.^8^ However, the main limitation of diagnosing root resorption with these methods is the compression of the three-dimensional anatomy into a two-dimensional image.^9^ Alternatively, CBCT imaging offers a three-dimensional visualization of the tooth and its supporting structures.^9^ Early diagnosis of root resorption is crucial during orthodontic treatment to identify teeth at risk of severe resorption.^22^ Therefore, it is essential to evaluate imaging methods capable of effectively diagnosing apical root resorption, particularly at its early stages.^10^ In the present study, we aimed to compare the accuracy of digital panoramic radiography, digital periapical radiography, and CBCT in diagnosing apical root resorption. Our findings demonstrated that CBCT imaging exhibited high sensitivity, specificity, and accuracy for detecting apical root resorption, followed by periapical and panoramic radiography, respectively. CBCT also showed a significantly greater ability to detect initial levels of resorption (up to 2 mm) compared to the other methods. For more advanced resorption (involving one-third of the root), periapical radiography performed similarly to CBCT. For extreme levels of resorption (involving half of the root), all three imaging methods performed comparably.

Requesting imaging exams is a common practice during orthodontic treatment, whether for planning, monitoring, or retention.^23^ Panoramic radiography is widely used due to its low radiation dose, ability to provide a complete view of the dental arch and supporting tissues, and convenience of image obtaining.^24^ Therefore, clinicians rely on panoramic radiography as the go-to method for assessing root length changes during orthodontic treatment.^24^ While digital panoramic radiography offers greater precision in diagnosing anatomical structures and produces less distorted images than the conventional method,^24^ previous studies demonstrated that it tends to overestimate the extent of root loss and faces limitations in detecting apical root resorption.^23,25^ In the present study, panoramic radiography demonstrated lower accuracy than periapical radiography and CBCT images, especially when detecting early-stage apical root resorption. This can be explained by the inherent two-dimensional nature of panoramic radiographs, which compresses the tridimensional anatomy of the roots, making subtle resorption changes harder to identify. In contrast, CBCT provides three-dimensional imaging, enabling better visualization of initial resorption, thus explaining its higher sensitivity in these cases. While also two-dimensional, periapical radiography offers a closer view of the apical region, which may account for its comparable performance to CBCT at more advanced stages of resorption. Consequently, while panoramic radiography is helpful for broader assessments, its limitations in detecting early root resorption make CBCT or periapical radiographs more reliable for detailed diagnosis.

Periapical radiography offers a more focused view with greater detail and less distortion over a smaller coverage area than panoramic radiography.^24^ Digital periapical radiography provides additional benefits, such as reduced radiation exposure, enhanced image detail, and the ability to manipulate or edit images.^8^ However, previous research has not definitively determined whether it outperforms conventional radiography in diagnosing root resorption.^8,26,27^ In the present study, periapical radiography exhibited sensitivity and specificity rates of 91.1% and 86.7%, respectively. These results are consistent with previous research, which reported sensitivity and specificity values ranging between 0.85 and 0.90.^9,28^ However, consensus regarding the high diagnostic performance of this imaging modality remains elusive. Other studies have reported sensitivity values below 80% and specificity below 70% for digital periapical radiography.^8,29^ A key distinction in our study was the ability of examiners to magnify images up to 50% of their original size, an option not consistently applied in other investigations. This capacity for image magnification is a clear advantage of digital radiography and was implemented in the present study to replicate clinical conditions. Digital magnification likely contributed to the improved detection of apical root resorption in this study, helping to minimize the performance gap between periapical radiography and CBCT.

In the present study, CBCT demonstrated high sensitivity, specificity, and accuracy in detecting apical root resorption. Its diagnostic superiority can be attributed to advanced imaging capabilities, providing three-dimensional views and submillimeter slicing.^28^ These detailed images enable clinicians to assess tooth structures from multiple angles, facilitating the identification of signs of root resorption.^28^ Previous studies support these findings, consistently showing that CBCT outperforms periapical and panoramic radiography, with sensitivity and specificity values exceeding 0.90.^9,28,29^ However, despite its diagnostic advantages, the routine use of CBCT for detecting root resorption is not recommended due to the potential risks associated with increased exposure to ionizing radiation.^30^ Following the "as low as reasonably achievable" (ALARA) principle, CBCT should be reserved for clinical scenarios in which two-dimensional imaging provides insufficient information for safe decision-making.^27,30^ These scenarios include: a) cases in which early but progressive root shortening (≤2 mm) is suspected on periapical radiographs but cannot be confirmed due to image overlap or anatomical limitations; b) patients presenting with atypical resorption patterns, such as unilateral or tooth-specific rapid progression; c) situations in which treatment modification (e.g., force reduction, interruption of orthodontic mechanics, or change of biomechanics) depends on precisely determining the extent of root loss; and d) cases with complex root morphology or pre-existing structural alterations where the apical region cannot be reliably visualized on periapical radiographs.^27,30^ In these contexts, CBCT can provide essential three-dimensional detail to guide timely intervention while still adhering to ALARA-based justification.^27^

Sensitivity and specificity are correlated measures and should not be considered in isolation when evaluating diagnostic accuracy.^31^ Previous reviews have emphasized the importance of considering both sensitivity and specificity when interpreting the accuracy of radiographic and CBCT methods for detecting root resorption.^10,27^ However, these measures may not fully capture an imaging method's diagnostic performance, as they do not account for its ability to discriminate between different degrees of resorption severity.^10^ To provide a more accurate assessment, the present study employed ROC curves to evaluate the overall performance of imaging methods as diagnostic tools. ROC curves allow for the simultaneous consideration of sensitivity and specificity across various thresholds, offering a more reliable measure of how well an imaging method can distinguish between different levels of root resorption.^31^ This is particularly important in orthodontic treatment, where root resorption is often less than 2 mm in extent.^32^ Given this, imaging methods must be able to detect these small changes to facilitate timely and effective clinical intervention.

In the present study, CBCT proved especially valuable in detecting early or milder stages of apical root resorption, up to 2 mm. For more advanced root resorption, involving up to one-third of the root, periapical radiography performed similarly to CBCT but was superior to panoramic radiography. For more severe levels of resorption, the three methods performed comparably. These findings are consistent with previous investigations, which reported a higher proportion of correct interpretations in cases with more advanced resorption, even when using different statistical comparison methods.^8,33^ Clinically, these findings highlight the need for a selective approach when choosing an imaging method. CBCT should be reserved for cases where early detection of mild resorption is critical or when other imaging methods, such as periapical radiography, raise concerns but require further investigation.

This study has some limitations that should be considered when interpreting the results. First, the images were obtained with the skulls and jaws in a static position, which does not fully replicate clinical conditions, where slight patient movement may affect image quality and diagnostic accuracy. Second, apical resorption was mechanically simulated using diamond burs, resulting in defects with relatively sharp and regular margins. This differs from the irregular contours and variable mineral density typically observed in biological resorption, which may influence image perception and diagnostic performance. Nevertheless, this approach was adopted to standardize the samples and clearly differentiate the various levels of resorption, thereby improving comparability with previous studies and enabling more controlled assessments. Third, the teeth were extracted and repositioned in their sockets to simulate resorption, which differs from real clinical scenarios in which root resorption is usually accompanied by periapical bone remodeling. Additionally, although crushed bone was used to fill the sockets in an attempt to simulate trabecular bone, its radiographic density and structural pattern may not fully reproduce those of natural alveolar bone. Finally, a methodological aspect that should be considered is that exact matching of CBCT cross-sectional slices between T1 and T2 was not always achievable due to inherent limitations in specimen repositioning and image reconstruction. To address this, slice selection was guided by anatomical landmarks, and minimal adjustments were performed by an experienced radiologist to ensure consistent evaluation of the same anatomical region. Although this approach may have resulted in slight differences in slice positioning, these were standardized and unlikely to have introduced systematic bias, thus preserving the validity of the diagnostic comparison.

The present study followed rigorous experimental procedures, as all assessments were performed under controlled *in vitro* conditions. Such conditions eliminate variables inherently present in clinical practice, including patient movement, anatomical variability, soft-tissue superimposition, and heterogeneous bone density. These factors may reduce the diagnostic performance of imaging modalities *in vivo* compared with the accuracy observed in our laboratory setting. These aspects should be considered when extrapolating the present findings to clinical settings, as such transposition must be done with caution. Further *in vivo* studies are needed to determine the extent to which these results reflect diagnostic performance in daily clinical practice.

## Conclusions

CBCT imaging presented high sensitivity, specificity, and accuracy in detecting apical root resorption, followed by periapical and panoramic radiography, respectively. Furthermore, CBCT exhibited a significantly greater ability to identify milder resorption levels (up to 2 mm) than the other methods. For more advanced resorption (involving one-third of the root), periapical radiography performed comparably to CBCT. For extreme levels of resorption (involving half of the root), all three imaging methods performed comparably. Therefore, CBCT appears to be the most suitable method for early ARR detection, whereas periapical and panoramic radiography are sufficient for diagnosing the advanced stage.

## Data Availability

The authors declare that all data generated or analyzed during this study are included in this published article.
